# In-season performance of European Union wheat forecasts during extreme impacts

**DOI:** 10.1038/s41598-018-33688-1

**Published:** 2018-10-18

**Authors:** M. van der Velde, B. Baruth, A. Bussay, A. Ceglar, S. Garcia Condado, S. Karetsos, R. Lecerf, R. Lopez, A. Maiorano, L. Nisini, L. Seguini, M. van den Berg

**Affiliations:** European Commission, Joint Research Centre, Via E. Fermi 2749, 21027 Ispra, Italy

## Abstract

Here we assess the quality and in-season development of European wheat (*Triticum spp*.) yield forecasts during low, medium, and high-yielding years. 440 forecasts were evaluated for 75 wheat forecast years from 1993–2013 for 25 European Union (EU) Member States. By July, years with median yields were accurately forecast with errors below ~2%. Yield forecasts in years with low yields were overestimated by ~10%, while yield forecasts in high-yielding years were underestimated by ~8%. Four-fifths of the lowest yields had a drought or hot driver, a third a wet driver, while a quarter had both. Forecast accuracy of high-yielding years improved gradually during the season, and drought-driven yield reductions were anticipated with lead times of ~2 months. Single, contrasting successive in-season, as well as spatially distant dry and wet extreme synoptic weather systems affected multiple-countries in 2003, ’06, ’07, ’11 and 12’, leading to wheat losses up to 8.1 Mt (>40% of total EU loss). In these years, June forecasts (~ 1-month lead-time) underestimated these impacts by 10.4 to 78.4%. To cope with increasingly unprecedented impacts, near-real-time information fusion needs to underpin operational crop yield forecasting to benefit from improved crop modelling, more detailed and frequent earth observations, and faster computation.

## Introduction

**Figure 1:**
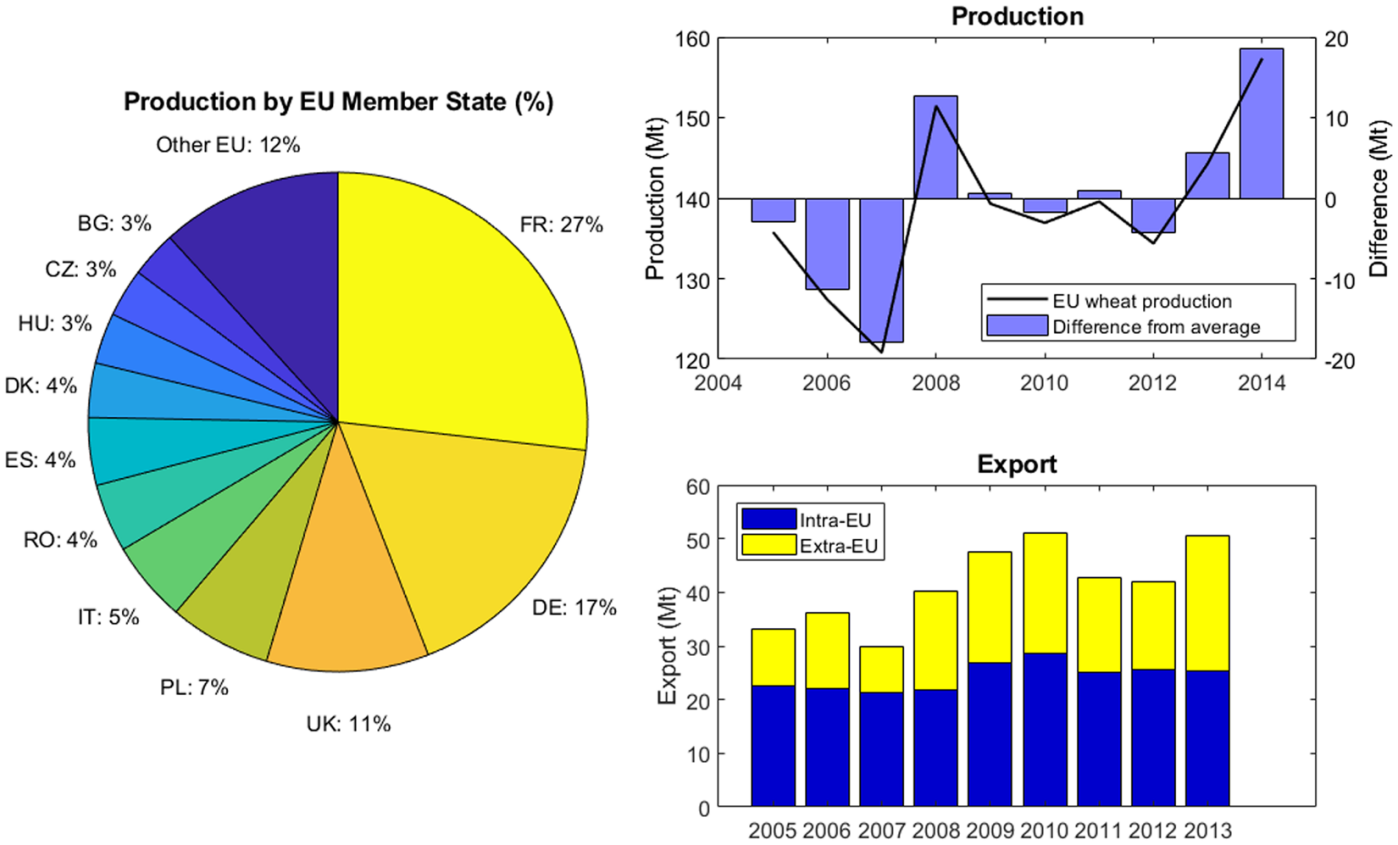
Production and export of common wheat (*Triticum aestivum*), durum wheat (*Triticum durum*) and spelt (*Triticum spelta*) by the EU. Contribution of each Member State to total EU wheat production over the 2005–2014 period (pie chart). Total annual EU wheat production from 2005 to 2014 compared to the average production over the entire period (upper right panel). Total annual EU wheat exports showing the share of intra- and extra-EU exports from 2005 to 2013 (lower right panel; no data available after 2013 as of 20 June 2017). Country codes are as follows: FR, France; DE, Germany; UK, United Kingdom; PL, Poland; IT, Italy; RO, Romania; ES, Spain; DK, Denmark; HU, Hungary; CZ, Czech Republic; BG, Bulgaria. Mt, million metric tonnes.

To have adequate, timely, and coherent information on expected crop production levels, the Joint Research Centre (JRC) of the European Commission (EC) forecasts crop yields and production across all European Union (EU) Member States (MS)^1–3^. The EU produces about 20% of global wheat^4^. Between 2005 and 2014, wheat production in the 28 countries that currently make up the EU (EU-28) ranged between 120.8 and 157.3 million metric tonnes (Mt) with a median of 138.1 Mt (Fig. 1). In 2012 31% of the wheat produced by MS was exported. From 2005 to 2014, intra-EU wheat export fluctuated between 21.1 Mt and 28.6 Mt, while between 8.7 Mt and 30.3 Mt of wheat was exported outside of the EU. Roughly, one-half of exports were destined for Algeria, Egypt, Morocco, Saudi Arabia, and Iran. Intra-EU export was more stable than extra-EU export. For example, wheat production fell by 5% in 2007 compared to 2006, but extra-EU export decreased by 38% (Fig. 1). To secure cereal supply and maintain national food balances, information on expected EU crop production levels is thus of direct relevance not only to EU MS but also to countries in North Africa and the Middle East.

Timely information on expected crop production is gaining importance as commodity markets are becoming more and more interconnected^5^. In a changing climate, impacts across large crop production areas due to increasingly variable or extreme weather and pest outbreaks can create knock-on effects that may affect food prices and availability elsewhere^6–8^. Several governmental and private sector institutes make crop production and commodity supply estimates at the national level^9^. These estimates rely on a variety or combination of qualitative and quantitative techniques for monitoring and forecasting, including field surveys, farmer inquiries, expert analysis, crop models, earth observation, or statistically based methods^10^. Estimates allow producers, exporters, importers, traders, and companies to make informed decisions across sectors covering raw materials, manufacturing, and sales and services. Sharing crop yield forecast information could help to mitigate market volatility, e.g., through a platform like the Agricultural Market Information System (AMIS; www.amis-outlook.org) launched in 2011 in response to the 2007/2008 food crisis.

While automated forecasting approaches have been assessed thoroughly^2,9,11–13^, most published forecasts rely on interpretation by analysts. This is the case for USA crop production forecasts by the National Agricultural Statistics Service (NASS)^14^ and the Foreign Agricultural Service (FAS, www.fas.usda.gov), and for EU MS forecasts by the JRC. Despite the importance of such forecasts, and a large and diverse stakeholder community, there are surprisingly few thorough quality assessments of published forecasts. An exception is the study by Egelkraut *et al*.^15^, who found larger forecast errors for USA yields of maize compared to soybean, although for both accuracy generally improved in successive months of the growing season, as more information about crop conditions and yields became available^15^. Other assessments have mainly been indirect and focused on commodity market reactions after the release of forecasts^16,17^. Here, we detail the performance of the JRC-EC national-level common wheat (*Triticum aestivum*) yield forecasts during low, median and high yielding years for each MS, identify when and where meteorological drivers had compounded effects on production losses, and discuss implications for wheat production forecasts.

## European Union crop forecasting

The JRC has forecast national-level EU crop yields and production since 1993. The crop yield forecasts are publicly available and published monthly during the growing season in the Monitoring Agricultural Resources (MARS) Bulletin with a description of dominant agro-meteorological conditions. Crop production forecasts are obtained by multiplying the crop yield forecast by the sown area, information provided by each MS to Eurostat, the statistical office of the EU. The crop production forecasts are used by the Directorate General of Agriculture and Rural Development, which then communicates production estimates to AMIS^1^. In a previous study, we assessed the accuracy, in-season, and year-to-year improvement of the MCYFS from 1993–2015^18^. When ranking the mean absolute percentage error (MAPE) for the end-of-campaign yield forecasts across all crops evaluated (soft wheat, durum wheat, grain maize, potato, rapeseed, sunflower, rapeseed) for each MS, the lowest median MAPE equalled 3.73% and was obtained for France, Europe’s largest producer^18^. The MAPE for soft wheat forecasts ranged from 3.11% for France (with a Mean Absolute Error – MAE – of 0.22 t ha^−1^) to 21.06% (0.38 t ha^−1^) for Portugal, with a median value of 7.18% (0.38 t ha^−1^) for Bulgaria. Operational forecasting systems aim to extrapolate the impacts of preceding and current conditions on expected future production as accurately as possible^19^. Monitoring crop status is therefore a key component of all operational crop forecasting systems. At the JRC, the MARS Crop Yield Forecasting System (MCYFS) facilitates monitoring of current crop conditions and forecasting of expected crop yield at harvest including country-level analyses. In the Methods section, a detailed summary of the steps of data analysis and interpretation done in the MCYFS needed to finalize and publish a yield forecast is presented. Statistical analysis is used to link past yield variability with predictors derived from interpolated meteorological observations, derived gridded agro-meteorological indicators, gridded crop model simulations^2^ – fed with interpolated observed meteorological data, which at the time of the yield forecast are extended by the 10-day forecast from the European Centre for Medium-range Weather Forecasting (ECMWF www.ecmwf.int), as well as remotely sensed observations. Statistical forecasting methods available to the analysts include trend analysis, regression analysis^20^ and similarity analysis based on principal component analysis (PCA) and cluster analysis^21^ (see Methods).

As the growing season starts, an acreage estimate and a trend yield forecast usually underpin the production forecast. Generally, little can be deduced of the impact of agro-meteorological conditions during the first weeks after seedling emergence, except those causing delays in sowing time or a significant decrease in sown area due to extreme weather. In such cases, possible interventions by the farmer (e.g. replanting) are crucial. During the overwintering period, wheat is exposed to frost damage risk, especially if a long warm weather anomaly is followed by an abrupt cold event. Once the wheat grows, and especially after heading, impacts of weather will become more discernible. The crop is especially sensitive to heat, drought stress, and water logging, during physiological processes such as flowering and grain formation^22^. As the crop nears maturity, with climate uncertainty becoming less of factor and data on cropped area more robust, the accuracy of the crop yield forecast reflects the accuracy of the model(s) used to make the forecast.

## Results

### Performance during extreme impacts

**Figure 2:**
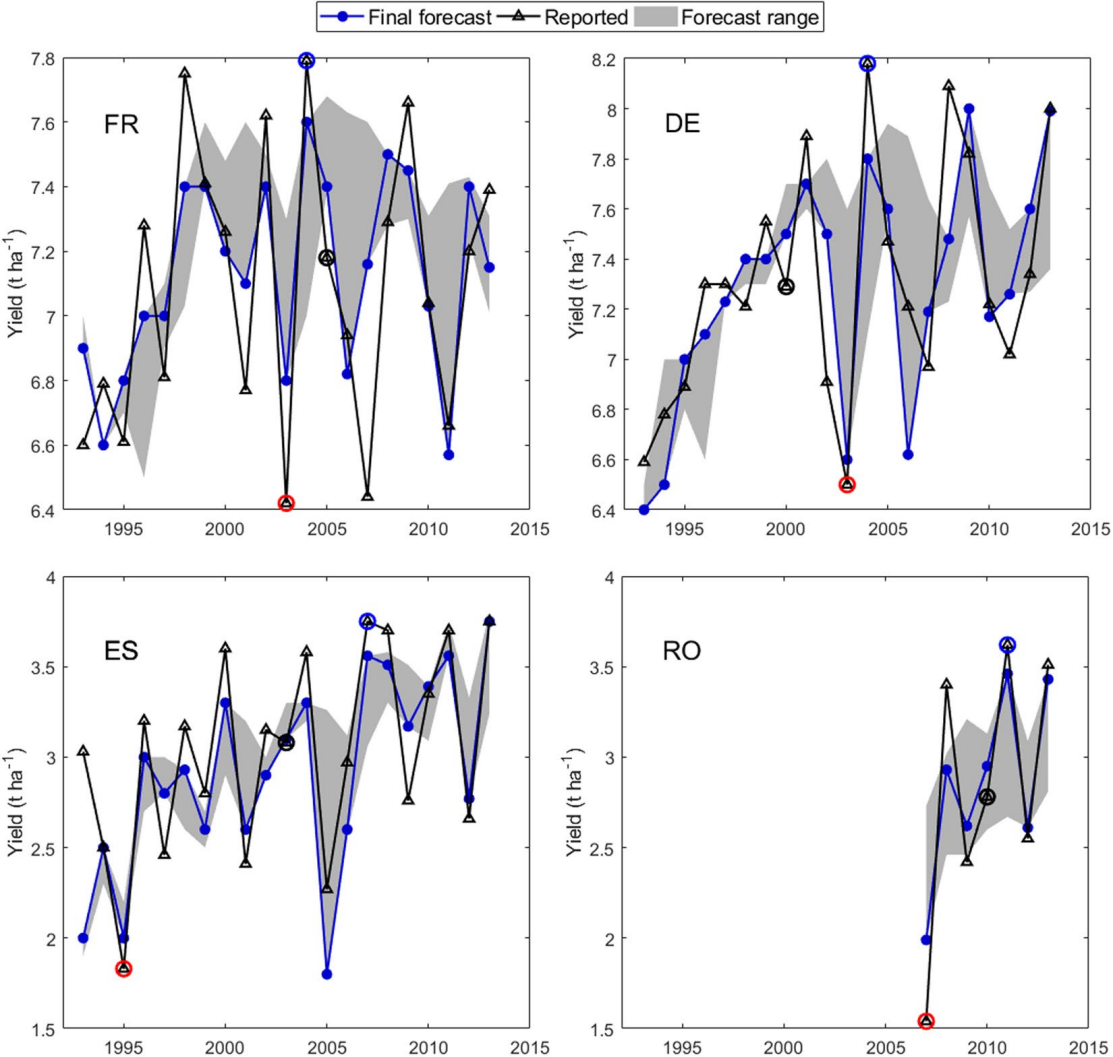
Final wheat yield forecast and reported wheat yields for four EU countries. Data are shown for France (FR), Germany (DE) and Spain (ES) from 1993 to 2013 and for Romania (RO) from 2007 to 2013. The grey shaded areas indicate the intraseasonal forecast range. The years with the lowest, median, and highest yield, that are analysed here are indicated respectively by red, black and blue circles. Note the different yield ranges on the y-axes.

Operational yield forecasting faces constantly changing challenges, such as dealing with the effects of newly developed crop management technologies, limited information on pests and diseases, and climate change. Climate change is affecting both the long-term trend and the interannual variability of yield^23^, as illustrated by the reported wheat yields, the end-of-campaign forecasts, and the intraseasonal forecasting range for four European countries (Fig. 2). Up to 2000, forecasts based purely on trends would have performed reasonably well in Germany and France. Since then, variability in yields in both countries has increased considerably, as has the range of yield forecasts during the growing season (Fig. 2). The forecasting system has coped with this larger yield variability, suggesting that the variability can be explained – at least partially – by interannual variability in agro-meteorological drivers. In Spain, both a trend and large interannual variability in yields are apparent, which were partially captured in JRC forecasts (Fig. 2). In Romania, forecasting has been done since 2007 when the country joined the EU and the accuracy of the forecasts has been high (Fig. 2).

**Figure 3:**
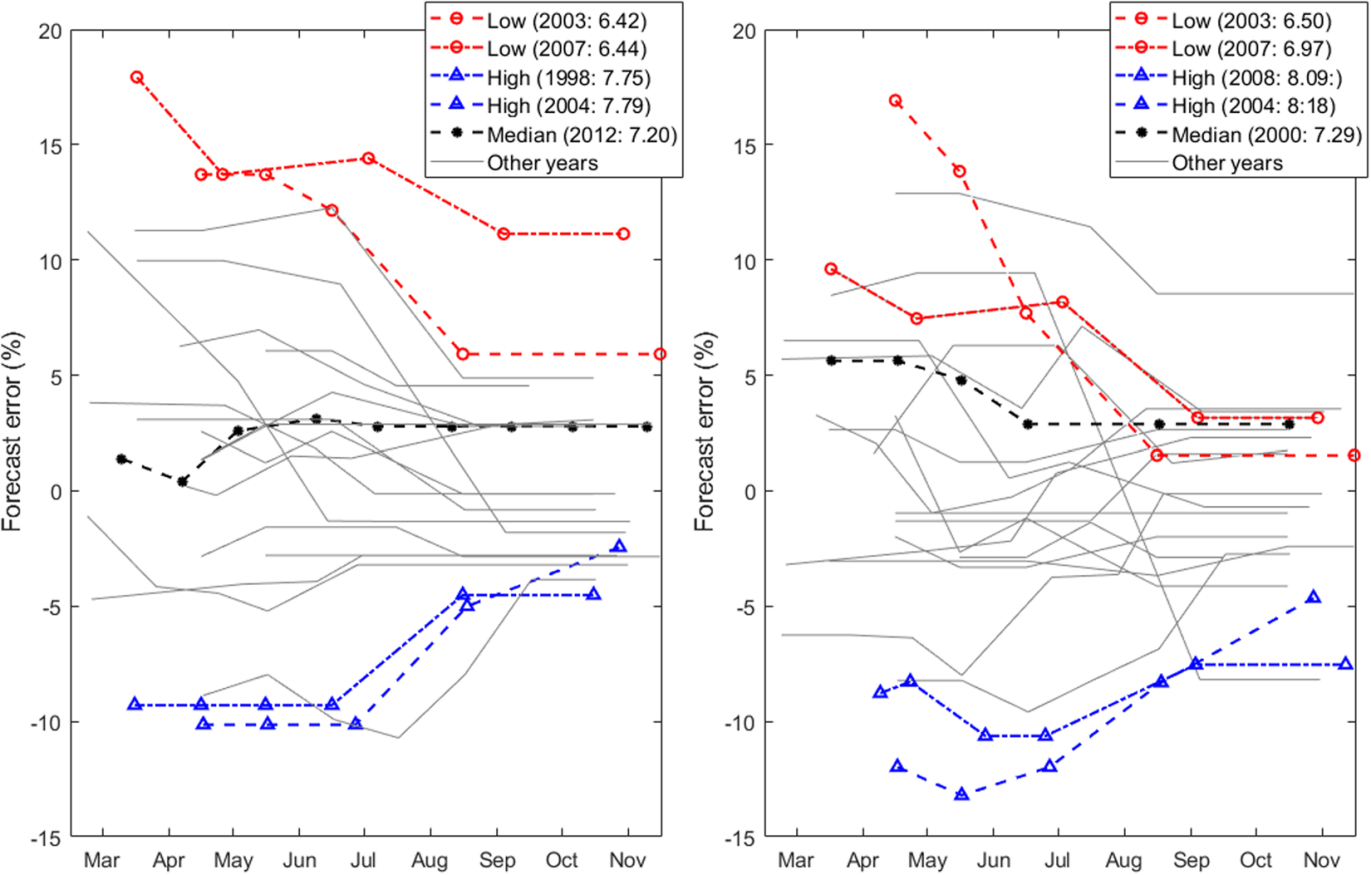
Changes in relative forecast error during the season for all wheat yield forecasts for France (left panel) and Germany (right panel) from 1993 to 2013. Numbers in brackets in the keys indicate the year and the reported yield in metric tonnes ha^−1^ for low, median and high yielding years in each country.

We evaluated wheat forecasts made for 25 MS in the 1993–2013 period focussing on the years that resulted in the lowest, the median, and the highest yield recorded in each MS (see Supplementary Table 1). During this period for each of the 25 MS, the forecasts in the year with the lowest, the year with the median, and the year with the highest reported yield were selected for further analysis. Figure 3 shows intraseasonal changes in the relative wheat yield forecast error for France and Germany (see Methods) for each season from 1993 to 2013. During the extreme years, as the seasons progressed, forecast errors converged to lower values. The years that resulted in the median yield for this period – 2012 in France and 2000 in Germany – are also the years when the initial trend forecast at the start of the growing season remained relatively unaltered. During 2003 and 2007, low yielding years for both Germany and France, forecasts were gradually lowered as the season progressed, but remained above the yield ultimately reported. In 2007, the wheat yield forecast in France was overestimated by more than 10%. During the high yielding years of 1998 and 2004 for France and 2008 and 2004 for Germany, forecasts were gradually increased but yield was still underestimated by about 5%.

**Figure 4:**
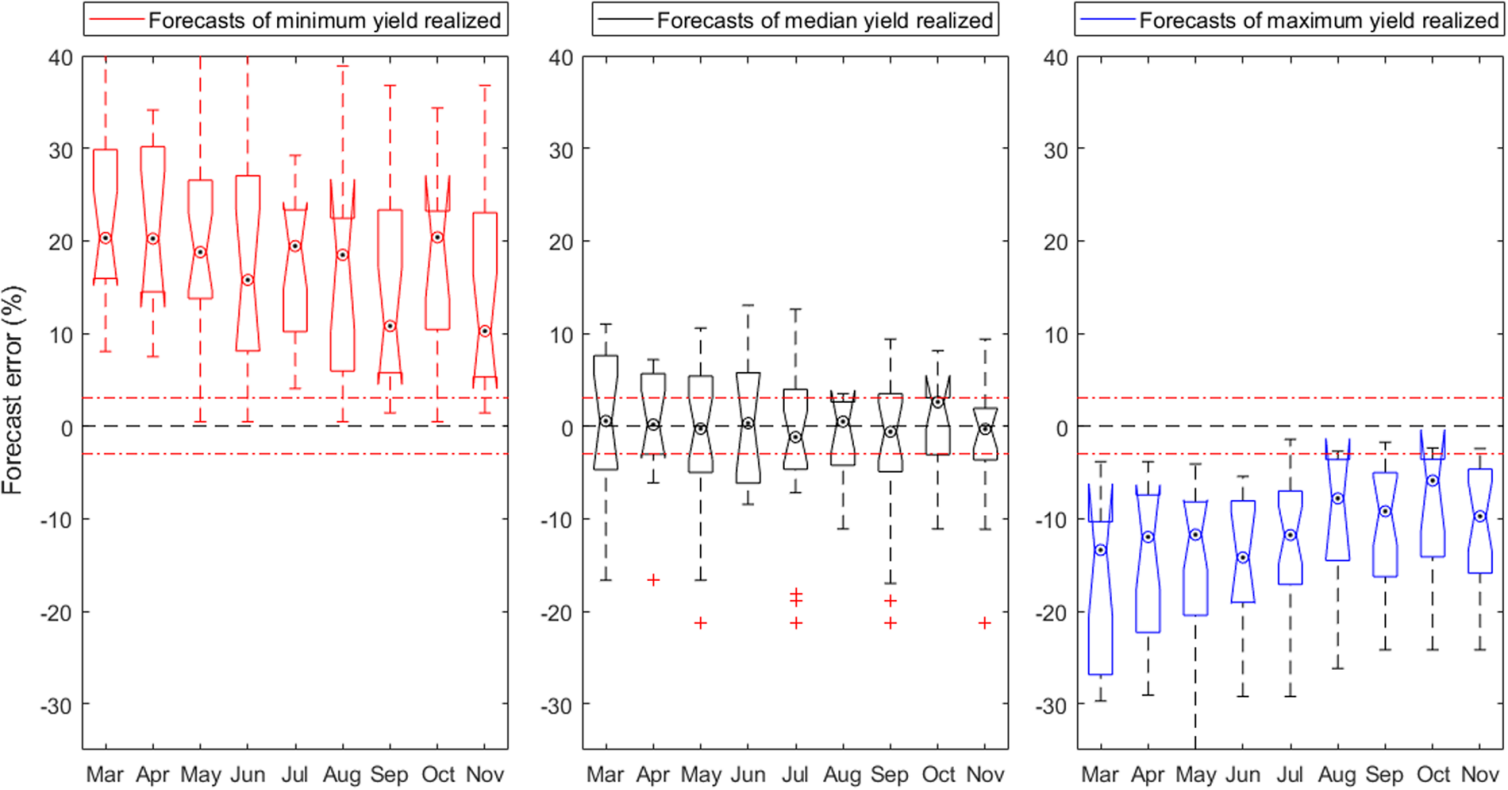
In-season development of forecast error for all Member States in years that resulted in minimum (left panel), median (middle panel) and maximum (right panel) common wheat yields in the 1993–2013 period. In each box, the dot in the white circle indicates the median, and the bottom and top edges of the box indicate the 25th and 75th percentiles, respectively. Notches display the variability of the median between samples. The width of a notch is computed so that box plots whose notches do not overlap (as above) have different medians at the 5% significance level. The significance level is based on a normal distribution assumption. The whiskers, defined as 1.5 times the interquartile range away from the top or bottom of the box, extend to the most extreme data points not considered to be outliers (red crosses). The red dashed lines indicate + and – 3%.

Being able to be anticipate crop failure, bumper years, or just average production levels is crucial. We evaluated the performance of the forecasting system from 1994 to 2013 in years when minimum, median and maximum wheat yields were reported for all MS, tracing the changes in relative forecasting error during the season (Fig. 4 and Supplementary Fig. 1). Median yields seem easy to forecast (Fig. 4, middle panel), and the variability in forecasts decreased slightly as seasons progressed. Forecasts of average weather and crop growing conditions by extrapolating the predominant trend in yields is the first basic requirement of the system. However, it is important to realize that these are *ex-ante* forecasts, and predicting average yielding years is not necessarily easy, especially since median yields do not always result from ‘normal’ or ‘average’ meteorological conditions. However, since crop yield integrates the cumulative effect of weather variability throughout the season, and crops can recover from impacts^24^; linking the impact of a single weather event to crop yield is not necessarily straightforward.

For instance, in 2010 in Hungary, early crop growth conditions were very beneficial leading to high forecasts, but prospects deteriorated later in the season due to over-wet conditions, finally resulting in median wheat yields. In years with high yields, March forecasts would underestimate final yields by around 12% to 30% (Fig. 4, right panel). As the seasons progressed, the forecast error was gradually reduced as processes beneficial for crop growth persisted (Supplementary Table 3) and the yield forecast increased incrementally. By August, the median of the forecast error associated with high yields was below 8%. In years with low yields, in-season improvements in the forecast error are less obvious. For water-limited countries such as Spain, Italy and Hungary, the lead times to predicting low yields were considerable (Supplementary Fig. 2). Yield losses due to dry conditions were forecast ~2 months in advance. This is because the crop model predictors used in the statistical forecasting accurately represent water stress^25^. By contrast, in countries where low yields do not tend to be caused by drought (e.g. the Netherlands), extreme impacts can result from excess precipitation during the later stages of the growing season, or even during the harvest. For instance, lodging of wheat due to heavy rain and/or heavy wind, can lead to a near-complete crop failure. Nevertheless, forecasts for France, Germany, United Kingdom, Poland and Italy (see Fig. 1), the five largest wheat producing MS accounting for about two-thirds of total EU wheat production, did anticipate the magnitude of the yield loss and gain in low and high yielding years, respectively (Supplementary Fig. 3). A drastic and unforeseen loss of wheat yield occurred in France 2016, driven by compounding extremes of abnormally warm temperatures in late autumn and abnormally wet conditions the following spring^26^. The particular circumstances during 2016 strained forecasting systems. The expectation that such conditions will occur more frequently in the future^26^, highlights the need for carefully assessing and improving operational crop forecasting capacity.

### Linking drivers with impacts

The country-specific analysts evaluated the drivers causing the low or high yield in the selected years (respectively Supplementary Table 2 and 3; and see Methods). When examining the reasons for the low yields reported (see Supplementary Table 2), we found that drought, drought in combination with a heat wave, drought in combination with excessive rain, or excessive rain, caused severe wheat production losses during the periods analysed. Without taking account of the magnitude of production losses, around four-fifths of the lowest-yielding years at MS level had a drought (or hot) driver, a third had a wet driver, while a quarter had both (Supplementary Table 2).

Years when bordering countries were impacted by the same large-scale cross-border synoptic weather systems could be identified. Such large-scale weather systems affected even groups of countries in five different years (Supplementary Table 2). In 2003, persistent drought and heatwaves across Europe lowered wheat production in France, Germany and Italy. In 2007, EU wheat production fell due to different meteorological extremes, with excessive rainfall negatively affecting wheat production in France, Germany, Belgium and the Netherlands, while drought across Hungary and Bulgaria caused production losses there. Shorter (heavy rainfall) and longer term (drought) processes therefore impacted yield in western and eastern Europe, respectively, in the same year. Similarly, in 2012 excessive rain led to low yield in Ireland and the UK, whereas a heatwave led to very low yields in Austria and the Czech Republic. In 2006, a large drought affected wheat production in Latvia, Lithuania and Poland. In 2011, Denmark, Estonia and Sweden suffered first from drought between March and mid-May when wheat stems were elongating during the vegetative phase, and then from excess rain between July and mid-August, during the crop’s reproductive phase and until harvesting. Of course, in countries where the main wheat producing regions border each other yield responses to the same large-scale weather anomaly may also be similar as they often share the same physiographic (e.g. soil quality) conditions, and comparable levels of intensification and agricultural technology. This is the case for the south of Romania and the north of Bulgaria that share the fertile Danube plains, the north of France and Belgium, and Austria and the Czech Republic.

### Implications for EU wheat production forecasts

In the EU single market from 2009 to 2013, wheat production levels averaged 129 Mt, and JRC-MARS forecasting errors were within −2.91 Mt to +1.97 Mt, that is −2.3% to +1.5%. For a climatic impact to translate into a detectable reduction of EU-28 production levels, it needs to affect multiple countries, including the larger producers. Clear examples of years when this is the case are 2003 and 2007. In 2003, 2006, 2007, 2011, and 2012, the years with multiple-country impacts, total losses in the countries impacted were between 0.1 Mt and 8.1 Mt compared to previous 5-year averages (Table 1). Larger harvested areas compensated for the yield impact of the 2011 drought in Denmark, Estonia and Sweden, compared to the previous five years. In relative terms, grouped losses ranged from 1.7% in 2011 to 25.3% in 2007. In these years, the losses induced by extreme weather accounted for between 0.1% and 7.9% of total EU-28 wheat production. In 2011, no overall EU-28 wheat production losses occurred compared to the previous 5 years. In 2012, losses equalling 2.9 Mt from Austria, the Czech Republic, Ireland and the UK were partially compensated for by production elsewhere, so EU-28 production was down by just 2.6 Mt. Extreme conditions in 2003, 2006 and 2007 contributed between 40.1% and 72.2% of total EU-28 losses. June production forecasts just before harvest are generally the most important ones for stakeholders. However, for the countries affected by the large-scale extreme weather events, June forecasts underestimated losses by between 10.4% and 78.4%. The end-of-campaign forecasts were better, but production was still overestimated by between 4.2% and 47.9% (Table 1).

**Table 1:** Wheat production losses, contribution to total EU-28 losses and forecast errors in years with extreme weather impacts affecting multiple countries.

	Grouped loss (Mt)*	Grouped loss (%)*	Contribution to EU-28 loss (%)*	Proportion of total EU-28 production (%)	June production forecast error (%)	End-of-campaign production forecast error (%)
2003 (DE, FR)	8.1	14.3	44.1	7.9	10.4	4.2
2006 (LT, LV, PL)	2.3	21.5	72.2	2.0	22.7	7.0
2007 (BE, NL, BG, HU, RO)	4.1	25.3	40.1	3.6	48.0	37.4
2011 (DK, EE, SE)	0.1	1.7	*No EU-28 loss*	0.1	12.8	6.6
2012 (AT, CZ, IE, UK)	2.9	13.4	110.9^†^	2.3	78.4	47.9

Countries that were not part of the EU during the periods considered were included in the EU-28 totals during the time periods analysed. Country codes are as follows: DE, Germany; FR, France; LT, Lithuania; LV, Latvia; PL, Poland; BE, Belgium; NL, Netherlands; BG, Bulgaria; HU, Hungary; RO, Romania; DK, Denmark; EE, Estonia; SE, Sweden; AT, Austria; CZ, Czech Republic; IE, Ireland; UK, United Kingdom. Mt, million metric tonnes.

*Compared to previous 5-year average.

^†^Loss compensated elsewhere.

## Discussion

We found that single weather events across countries (e.g. drought in Latvia, Lithuania and Poland in 2006), contrasting successive in-season extremes (e.g. dry and wet in Denmark, Estonia and Sweden in 2011), or spatially distant extremes (e.g. dry in Bulgaria, Hungary, Romania and wet in Belgium, Netherlands and France in 2007) contributed >40% to total EU losses in those years. Since large weather systems can affect crop production in several countries during the same year, more in-depth investigation into the behaviour, similarity, drivers, and predictability of reported yield statistics in cross-national bordering regions is needed. This is especially important if there are disparities in data availability and quality between bordering countries.

The results presented here of synoptic weather systems affecting multiple countries at the same time illustrate the necessity of international monitoring and forecasting of crop production, as is done in the EU. However, the lead-time and quality of forecasts during extreme impacts needs to be improved. So far, the skill of seasonal dynamical weather forecasting is rather weak or even non-existent across Europe^27^, unlike, for example forecasting of the impact of El Niño-Southern Oscillation on Australian agriculture. Recently, advances have been made in linking crop yield anomalies to large-scale weather patterns^28^ and benefits should be had from continuously improving skill in medium and extended-range^29^, and even seasonal forecasts^27^. Speculatively, it is intriguing to consider whether contrasting extremes across Europe might (sometimes) relate to predictable large-scale circulation patterns. In 2007, heavy rainfall lowered yields in northwestern Europe, while the east suffered from a large-scale drought. Similarly, excessive rain affected Ireland and the UK in 2012, while heatwaves lowered yields in Austria and the Czech Republic.

The statistical analysis in the MCYFS forecasting machinery involves a degree of ‘art’, i.e. operator choice. This generally is the case, but it is especially true in years with extremes, simply because the statistical forecasting is limited by 1) capability of crop models to capture certain extremes, 2) the variability in the time series of past wheat yields not large enough to capture expected losses. Indeed, in certain years, when the statistical methods were not capable of forecasting a yield that the analysts would intuitively be expecting, so-called custom values (defined by the analysts outside of the statistical framework) have been used to forecast an extreme yield. For instance, in Luxemburg in 2011, with the lowest reported yield recorded at 5.54 t/ha from 1993 to 2014, a custom forecast was used to forecast 5.82 t/ha, which was the lowest forecast on record, but which was still above the realized yield. Conversely, for the highest yield on record at 3.62 ton/ha reported in Romania for 2011, a custom value was used to make a forecast of 3.46 ton/ha. The methods used by the analyst (e.g. trimmed 5-year average, 5-year average, trend (linear, logarithmic, polynomial), regression, or similarity analysis) have been tracked since 2006. Of course, there are also several examples of the analyst using the standard statistical approach, resulting in a bad forecast (e.g. not low, or not high enough, respectively for Ireland 2012, or for Italy 2012). Supplementary Table 4 summarizes an analysis evaluating the methods the analysts chose in the extreme years. This information was available for a total of 36 extremely low or high yielding country-year combinations (as this has only been recorded since 2006). Out of these 36, 9 forecasts were good (considering they had an error <5%). Out of these 9, 6 were based on similarity analysis, and 3 were customized values. In other words, a quarter of the forecasts could be considered ‘good’, and out of these good forecasts, a third was based on custom values.

Understanding the accuracy and development of yield forecasts as the growing season progresses is essential in deciding where in the system improvements are needed. In addition, this ensures the appropriate uptake and use of the forecasts^30^. Since crop forecasts can ultimately have such a wide-ranging influence on people’s livelihoods and access to food, especially in extreme situations, continual thorough assessment and communication of the accuracy of yield and production forecasts should be an essential part of any operational crop forecasting activity. Ideally, in an international context, assessments of crop yield forecasting performance need to be country and crop specific, distinguish between errors due to yield forecasts and acreage estimates, provide information with respect to minimum accuracy levels required, quantify lead times against accuracy requirements, and ultimately benchmark against other operational crop forecasting activities.

Forecasting agencies should also share lessons learned. Besides the JRC, government-backed initiatives include the NASS^14^ and World Agricultural Supply and Demand Estimates^17^ activities of the United States Department of Agriculture (USDA) and CropWatch, the crop monitoring system in China^13^, while the intergovernmental International Grains Council, (www.igc.int) provides – against payment – provisional figures during the season. Companies in the private sector also provide crop-forecasting services, and technological advances in recent years have led to increased activity focused on tailoring advice to farmers’ needs. In Europe, Copa-Cogeca, representing EU farmers’ and agricultural cooperative organizations, and Coceral – the European association representing the trade in cereals, rice, feedstuffs, oilseeds, olive oil, oils and fats and agro-supply – also forecast and release statements on expected grain and oilseed crop production levels. When USDA maize and soybean production forecasts were assessed, the performance of the USDA and two private agencies were comparable, with the relative forecast accuracy generally improving throughout the crop year^15^. Nevertheless, the mandates and motivations of organizations differ, as do the forecast methods they employ and the extent to which monitoring activities extend to quantitative yield and production forecasts. While field surveys and/or farmer interviews have traditionally informed regional or national crop yield estimates in many countries, recently, probabilistic approaches to forecast yields have been introduced^9,12^. Undoubtedly, the way forward will depend on improvements in observation systems, crop models, and weather forecasting. Improved understanding of processes behind extreme impacts will make it possible to improve crop models^31^. The aim of field-level forecasting systems is to facilitate crop management decisions to improve the performance of cropping systems^32^ while larger-scale forecasting systems – such as the one evaluated here – predict the output levels of regions or countries to anticipate expected fluctuations in crop production levels^19^. Ultimately, developments in technology, data access, and processing, may bring forecasting systems that operate at field level closer to those that operate at larger scales. For example, developments that foster open access to detailed geospatial reference datasets in combination with available high-resolution satellite images will enable a much better characterisation of impacts at field level^33^. These advances will also benefit from continual improvements in the skill of short-range or even seasonal weather forecasts^24,34,35^.

## Methods

### Forecast data

All the operational common wheat (*Triticum aestivum*) yield forecasts from 1993 to 2013 were collected from the printed MARS Bulletins^2^. This amounted to a total of 362 forecast years and 2450 in-season yield forecasts. From this dataset, for each of the 25 MS the forecasts in the year with the lowest, the year with the median, and the year with the highest reported yield were selected for further analysis (Supplementary Table 1). This resulted in 75 forecast years, and 440 in-season yield forecasts. For low, median, and high yielding years, respectively, 144, 142 and 154 forecasts were analysed. Values for yield and production used for the quality assessments were those communicated by the MS to Eurostat, published as of October 2015^36^.

### Area estimates

All operational national-level crop production forecasts require estimates of total sown crop acreage and forecasts of the end-of-season yield. The EU and national level forecasts by the JRC rely on estimates of total sown crop area provided by the MS to Eurostat. For each MS, forecasts are made for crops where the total crop area exceeds 10,000 ha within that country. In this study of operational production forecasting, crop acreage numbers were taken as given to focus on aspects related to the forecasting of crop yield.

### Linking drivers with impacts

During the period for which forecasts were made for each MS, the years with the lowest, median, and highest, reported wheat yield were selected for further analysis (Supplementary Table 1). The country-specific analysts analysed which drivers in each particular year could be associated with the lowest or highest reported yield (respectively Supplementary Table 2 and 3). The analysts revisited the years selected and identified the drivers by analysing the weather development during the growing season, reading the Bulletins of those years, as well as related scientific publications if available. In some cases, the analyst had been actively forecasting during the years selected. The drivers were not predefined. Resulting drivers for low yields were drought, heat waves, excessive rain, excessive soil moisture, disease and pest pressure, and other (see Supplementary Table 2). Analysts could also suggest combinations of drivers (e.g. drought and heat wave).

### Operational monitoring and forecasting system

**Figure 5:**
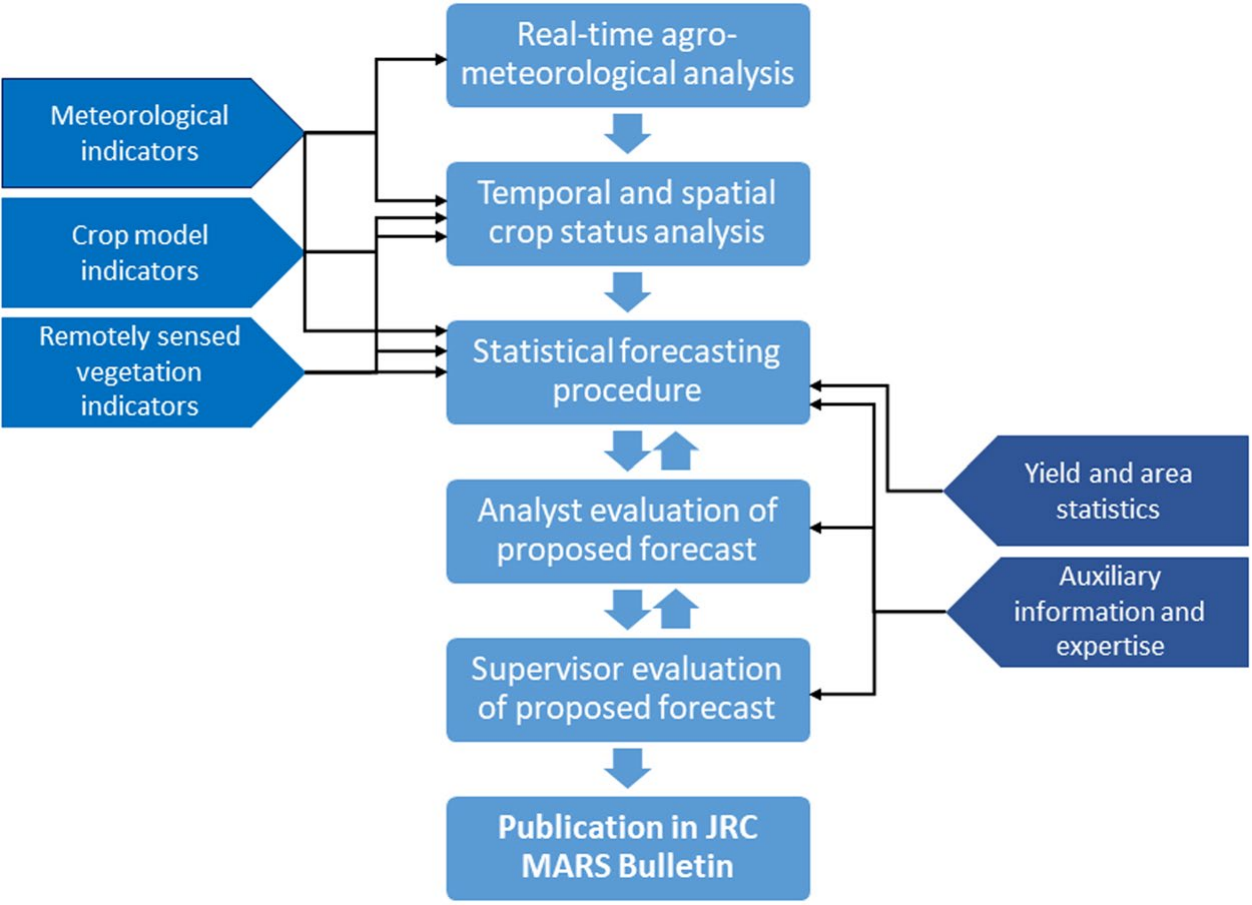
Flow chart illustrating the MARS-Crop Yield Forecasting System work flow. The middle block details the analysis steps, starting with real-time agro-meteorological analysis, and ending with the publication of the yield forecast in the JRC MARS Bulletin. This also includes the iterative evaluation of proposed forecasts, first by the analyst, and second by the supervisor. The left block lists the gridded spatial data infrastructures contributing quantitative data: the meteorological indicators, the simulated crop model indicators, and the remotely sensed vegetation indicators, with arrows indicating how they connect to the different steps of analysis. The indicators are used as predictors in the statistical yield forecasting. Trend analysis and selection is part of the statistical forecasting procedure. On the right side, the statistics on yields and area are essential input to the statistical forecasting procedure. Auxiliary information (e.g. news reports) and expertise of analysts are relevant for analyst choices with respect to the statistical forecasting procedure, as well as for the evaluation of the proposed forecasts.

The MCYFS^37^ underpins forecasts with country-level analyses by dedicated country analysts with various expertise (e.g. agronomy, meteorology, statistics or remote sensing). In Fig. 5, a flowchart summarizes the steps of data analysis and interpretation in the MCYFS needed to finalize and publish a yield forecast. The crop yield forecast procedures are based on analysing times series of national-level official yield statistics, by using different combinations of input; meteorological indicators based on observed and forecasted weather, simulated crop growth model indicators, remote sensing based indicators, all with a 10-day time step, and additional information sources and expert knowledge. An in-house developed software tool ‘the Control Board Forecast Manager’ or ‘CoBo’ is the engine of the statistical forecasting in the MCYFS. CoBo stores the indicators that are used as predictors in the statistical forecasting models. The analysts use CoBo for the statistical forecasting procedure. Supervisors use CoBo to manage the complete forecast analysis, and to collect and process all crop-country forecasts in order to produce coherent crop forecast tables, including aggregation to crop groups and to EU level.

During the first experimental season of the MARS forecasts, meteorological data from 700 weather stations were interpolated on a 50-km grid. The MCYFS has evolved over time and nowadays a 25-km grid is used with daily meteorological data coming from more than 4000 weather stations. Interpolated meteorological data date back to 1975 and in the ongoing growing season are extended with the 10-day ECMWF forecast. Quantification of the meteorological drivers is critical, as climate variability explains about a third of global crop yield variability^38^. Wheat crop modelling is based on the World Food Studies simulation model^39^ (WOFOST) fed with the meteorological data (including the 10-day ECWMF weather forecast) and implemented in the Bio-physical Model Applications Framework (BioMA) framework (http://bioma.jrc.ec.europa.eu/). The models are run on elementary simulation units that are defined as the intersection of the 25 km meteorological grid, and soil data from the European Soil Data Bureau. Once model outputs are aggregated at the national level, they are used as the 10-day predictors in the statistical analysis. Recently, remote sensing has been used to quantitatively forecast yields at the regional level^40^.

Statistical forecasting methods available include trend analysis (e.g. linear, logarithmic, etc.), regression analysis^20^ and similarity analysis (based on PCA and cluster analysis)^21^. The multiple-linear regression analysis requires the analyst to define a trend and identify predictors (e.g. WOFOST simulated water limited crop yield and development stage). The trend and predictors are input into the regression model to explain past yield variability. The derived regression model is then used to forecast the end-of-season yield, by feeding the regression model with the predictor values of the ongoing year. The similarity analysis relies on the identification of years where meteorological conditions and simulated crop model variables were most similar to those experienced in the current year. For the PCA and cluster analysis the predictors of all available years are used to establish a similarity matrix among the years. Yields that were reported in these similar years are then weighed by the distance in component-space to produce a yield prediction^21^. The statistical models used for the wheat forecasts from 1993 to 2013 analysed here, used predictors exclusively derived from the WOFOST crop model. Remote sensing was used qualitatively, e.g. by evaluating maps of fAPAR.

A range of forecasts can be produced, e.g. depending on choice of trend (e.g. period and type), or the choice of which indicators to use (e.g. simulated potential yield or soil moisture), as well as the time periods they cover (e.g. for dekad 18–20 and 19–22). After the analyst has made these choices, the resulting forecasts that are statistically significant, and have a logical causal relation between indicators and observed yield, are evaluated further. The analysts make the yield forecast, and evaluate different possible forecasts by themselves, but each analyst has discussions with the meteorological experts, the remote sensing experts, and the crop model experts. A group discussion is held 2–3 days before the Bulletin is published, where each analyst presents the agro-meteorological analysis of his or her countries, and the implications for each forecasted crop, and thus the underpinning reasons for the yield forecast. The agro-meteorological analysis, and the assessment of crop status, is also reported in the MARS Bulletin. Supervisors check the forecast, especially if the agro-meteorological analysis does not seem to be in line with the forecast made.

Theoretically, the geographic dimension of the forecast goes from the elementary modelling unit, to grid cells (25 km resolution), to the administrative regions. While indicators are systematically stored at grid resolution, and at different administrative levels, the final yield forecasts is given at a national level only. The aggregated EU yield is the results of weighting the national level yield forecasts with the most recent crop area data available. There is a continuing effort to evaluate and improve the indicators used^41^. This could refer to improved processing chains of remote sensing indicators, or region specific re-calibration of crop parameters of the WOFOST crop model^42^ in the CGMS. Whenever the configuration of the system changes (for instance after re-calibration of parameter values) all relevant indicators are re-processed to maintain database consistency.

## Analysis

To evaluate the in-season development of forecast performance, we calculated the absolute and percentage forecasting error. The absolute forecasting error *e*_*t,i*_ for year *t* and month *i* is defined by the difference between forecast *F* and reported yield *O* values communicated by the MS to Eurostat (2015):

1 $${{e}}_{t{,}i}={F}_{t,i}-{O}_{t}$$

Similarly, percentage error *pe*_*t,i*_ is defined as:

2 $$p{e}_{t,i}=\frac{({F}_{t,i}-{O}_{t})}{{O}_{t}}\times 100$$

## Electronic supplementary material

Supplementary Information
