# Supplementary material for: In-season performance of European Union wheat forecasts during extreme impacts
Source: Sci Rep. 2018 Oct 18;8:15420. doi: 10.1038/s41598-018-33688-1 (PMC6194012; doi:10.1038/s41598-018-33688-1)
Supplement: Supplementary file 1 — Supplementary Information [file 41598_2018_33688_MOESM1_ESM.pdf]

SUPPLEMENTARY INFORMATION

In-season performance of European Union wheat forecasts during extreme impacts

M. van der Velde[1*], B. Baruth[1], A. Bussay[1], A. Ceglar[1], S. Garcia Condado[1], S. Karetsos[1], R. Lecerf[1], R. Lopez[1], A. Maiorano[1], L. Nisini[1], L. Seguini[1], M. van den Berg[1]

[1]European Commission, Joint Research Centre, Via E. Fermi 2749, 21027 Ispra, Italy

[*] Correspondence to marijn.van-der-velde@ec.europa.eu

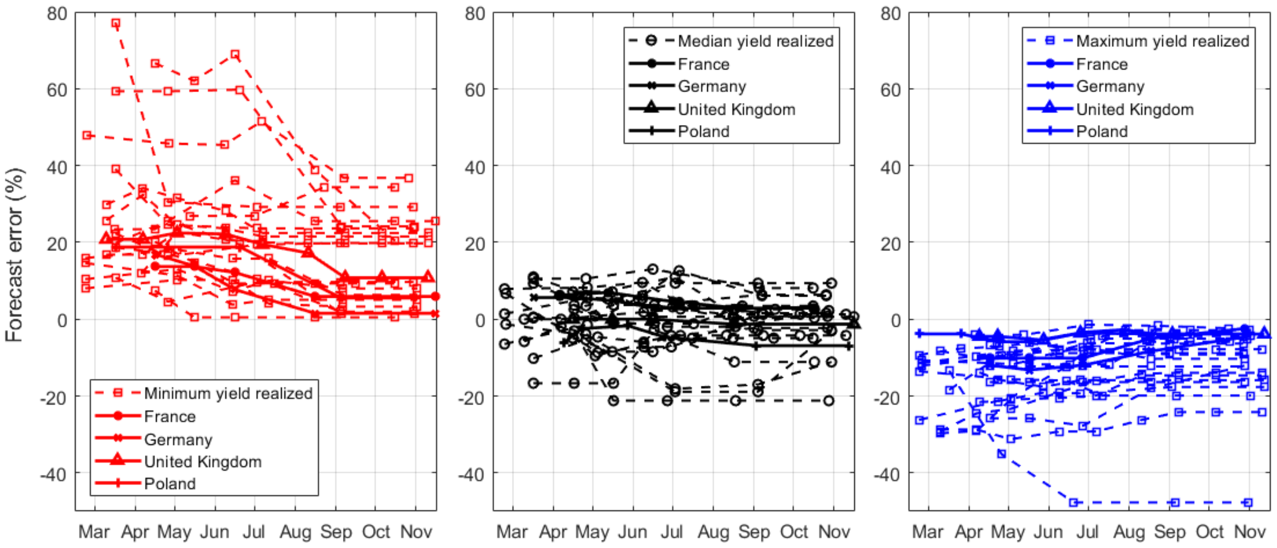

Supplementary Figure 1. Changes in common wheat yield forecast error for all Member States during the years that resulted in the minimum (left panel), median (middle panel) and maximum (right panel) yields in the 1993-2013 period. Data for France, Germany, United Kingdom and Poland, the four largest wheat producing Member States, are highlighted.

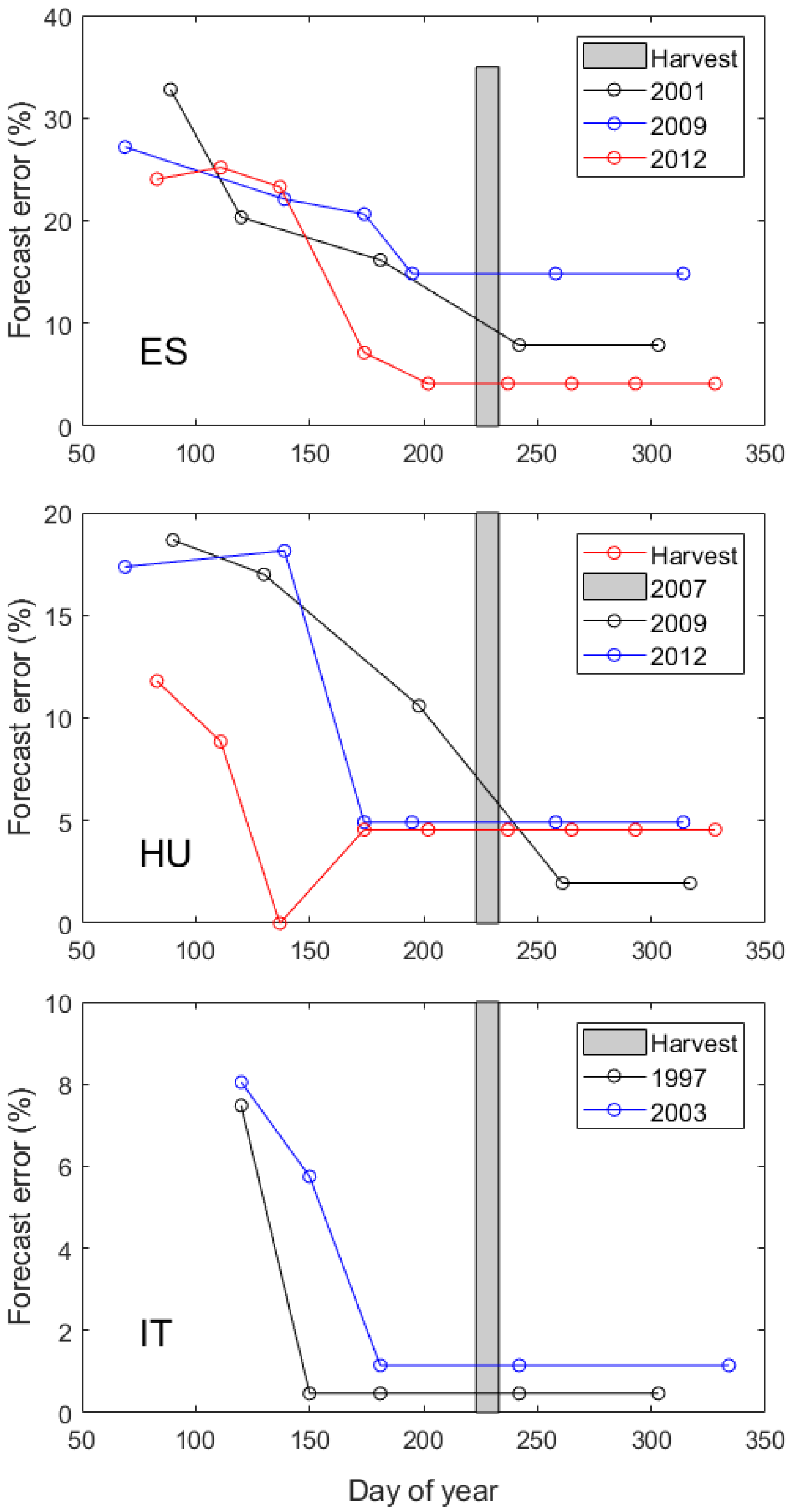

Supplementary Figure 2. Changes in wheat yield forecast error during dry years in Spain (2001, 2009, 2012; upper panel), Hungary (2007, 2009, 2012; middle panel) and Italy (1997, 2003; lower panel).

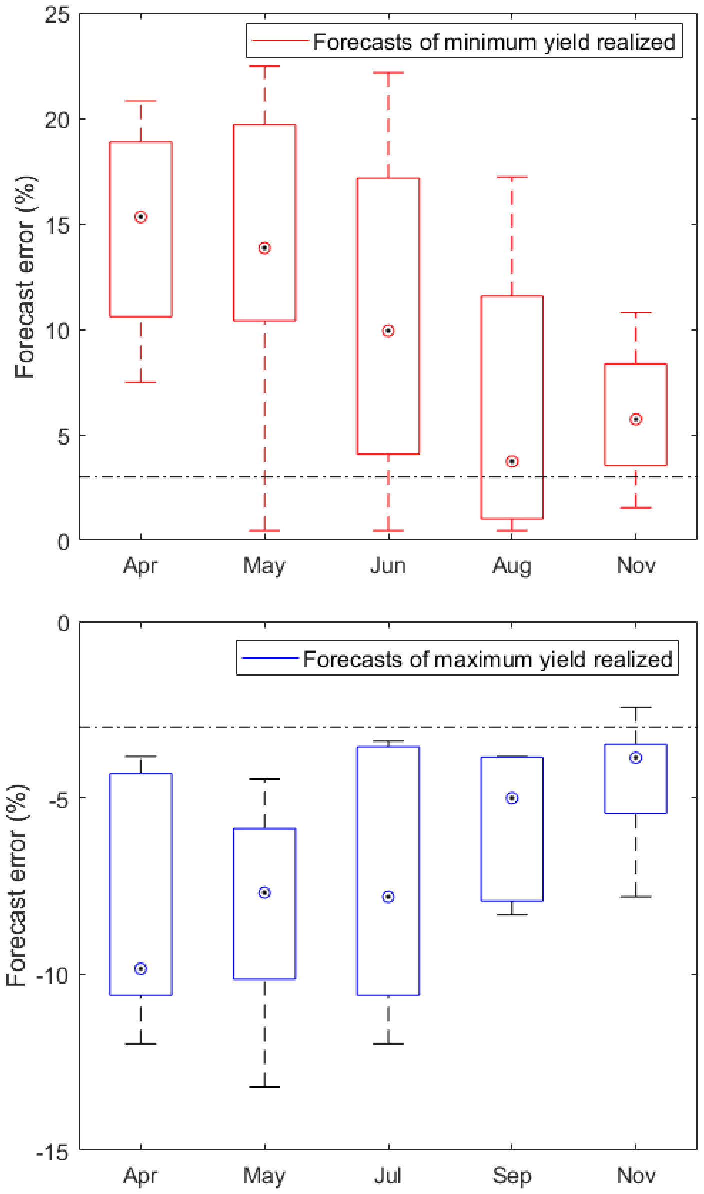

Supplementary Figure 3. In-season development of common wheat yield forecast error for five Member States in years that resulted in the minimum (upper panel; red) and maximum (lower panel; blue) yields during the 1994-2013 period. Months for which at least a forecast was done for 4 out of the 5 Member States of France, Germany, United Kingdom, Poland and Italy, which together account for >67% of total EU production, were considered in the analysis. In each box, the dot in the white circle indicates the median, and the bottom and top edges of the box indicate the 25th and 75th percentiles, respectively. The whiskers, defined as 1.5 times the interquartile range away from the top or bottom of the box, extend to the most extreme data points not considered to be outliers (red crosses). The dashed lines indicate + and – 3%.

Supplementary Table 1. The years of the lowest, median, and highest, reported yields over a given period of forecast years for each Member State. Standard country codes are shown. Tonnes are metric.

Code	Member State	Year (low)	Lowest yield (t ha^{-1})	Year (med)	Median yield (t ha^{-1})	Year (high)	Highest yield (t ha^{-1})	Period considered
AT	Austria	2012	4.19	1996	5.06	2004	5.98	1997-2013
BE	Belgium	2007	7.84	2005	8.41	2009	9.35	2004-2013
BG	Bulgaria	2007	2.2	2012	3.78	2013	4.2	2007-2013
CZ	Czech Republic	2012	4.32	2010	5.07	2008	5.85	2004-2013
DE	Germany	2003	6.5	2000	7.29	2004	8.18	1993-2013
DK	Denmark	2011	6.47	2004	7.14	2009	8.04	1994-2013
EE	Estonia	2006	2.74	2009	3.02	2012	3.97	2004-2013
ES	Spain	1995	1.83	2003	3.08	2007	3.75	1993-2013
FI	Finland	1999	2.16	2000	3.6	1996	4.08	1994-2013
FR	France	2003	6.42	2005	7.18	2004	7.79	1993-2013
GR	Greece	2001	2.35	1995	2.84	2011	3.32	1993-2013
HU	Hungary	2007	3.59	2006	4.07	2004	5.12	2004-2013
IE	Ireland	2012	7.2	2007	8.46	2004	9.92	1993-2013
IT	Italy	1997	4.28	2007	4.91	2012	5.89	1993-2013
LT	Lithuania	2006	2.36	2007	3.92	2012	4.78	2004-2013
LU	Luxembourg	2011	5.54	2010	5.96	2004	6.84	2004-2013
LV	Latvia	2006	2.78	2007	3.59	2012	4.37	2004-2013
NL	Netherlands	2007	7.21	2012	8.7	2009	9.29	2004-2013
PL	Poland	2006	3.25	2008	4.07	2013	4.43	2004-2013
PT	Portugal	2005	0.67	2004	1.65	2006	2.39	1993-2013
RO	Romania	2007	1.54	2010	2.78	2011	3.62	2007-2013
SE	Sweden	2011	5.38	2004	5.98	2005	6.35	1994-2013
SI	Slovenia	2009	3.87	2004	4.53	2012	5.44	2004-2013
SK	Slovakia	2010	2.8	2009	4.03	2008	4.87	2004-2013
UK	United Kingdom	2012	6.68	2011	7.8	2008	8.28	2000-2013

Supplementary Table 2. Meteorological factors that caused the lowest common wheat yields in the forecast periods analysed. Country-specific analysts determined the drivers for each year. Percentage below median yield is also indicated. Seven groups of countries for which low yields were driven by the same weather systems are shown in different colours, other countries are shown in white. Crosses indicate the relative importance of the driver. Details of country codes and forecast periods are given in Supplementary Table 1.

	AT	CZ	BE	NL	BG	HU	RO	DE	FR	DK	EE	SE	IE	UK	LT	LV	PL	ES	FI	GR	IT	LU	PT	SI	SK
	2012	2012	2007	2007	2007	2007	2007	2003	2003	2011	2011	2011	2012	2012	2006	2006	2006	1995	1999	2001	1997	2011	2005	2009	2010
Below median yield (%)	*-17.2*	*-15*	*-6.8*	*-17*	*-42*	*-12*	*-45*	*-10.84*	*-11*	*-9.4*	*-9.3*	*-10*	*-15*	*-14*	*-40*	*-23*	*-20*	*-41*	*-40*	*-17*	*-13*	*-7*	*-59*	*-15*	*-31*
Drought			X	X	XXX	XXX	XXX	X	XXX	XX	XX	XX			XX	XX	XX	X	X	XXX		XXX	X		
Heat wave	XXX	XXX			XX	X	XX	XXX	X										X					XXX	XX
Excessive rain			XXX	XXX						XX		XX	XXX	XXX							XXX				XXX
Excessive soil moisture																					X				
Disease and pest pressure																					X				XX
Other	XX*	XX*			X‖	X‖	X‖	X*,‡													XXX#			XX†	
Other								XXX§																	

*Shortening of grain filling period
†Storm with strong wind and hail
‡Delayed start to the season
§Reoccuring periods with low soil moisture
‖ Accelerated crop development
#Dry winter and spring conditions

Supplementary Table 3. Meteorological factors that caused the highest common wheat yields in the forecast periods analysed. Country-specific analysts determined the drivers for each year. Percentage above median yield is also indicated. Crosses indicate the relative importance of the driver. Drivers were identified and their importance estimated based on analysis by dedicated country experts. Colours indicated groups of MS affected by the same weather events. Drivers were identified and their importance estimated based on analysis by dedicated country experts. Details of country codes and forecast periods are given in Supplementary Table 1.

	AT	BE	BG	CZ	DE	DK	EE	ES	FI	FR	GR	HU	IE	IT	LT	LU	LV	NL	PL	PT	RO	SE	SI	SK	UK
	2004	2009	2008	2008	2004	2009	2012	2007	1996	2004	2011	2004	2004	2012	2012	2004	2012	2009	2013	2006	2011	2005	2012	2008	2008
Above median yield (%)	*18.18*	*11.2*	*11.1*	*15.4*	*12.2*	*12.6*	*31.5*	*21.75*	*13.3*	*8.5*	*16.9*	*25.8*	*17.3*	*20*	*21.9*	*14.8*	*21.7*	*6.78*	*8.85*	*44.8*	*30.2*	*6.19*	*20.1*	*20.8*	*6.15*
Beneficial radiation levels		XX				X	XX		XX				X		XX	X	XX	XX				X			
Beneficial heat accumulation	XX	X		XXX	XX	XX		X					X	XXX		X		X		X				XXX	X
Beneficial precipitation distributior	XXX	XX	XXX	XX	XX	XX		X		XX	XXX	XXX	X	XXX		XX		XX	X	X	XXX	XX	XXX	XX	X
Cold but not too cold winter					XX		XX		XX			X		XXX	XX		XX		XX						
Other			XX*		XX†																				
	*Mild early spring																								
	†Good conditions during flowering																								

Supplementary Table 4. Summary of methods used to forecast extreme low and high yields.

	**Forecasts of extreme low or high yields**		**Methods**		
	Total forecasts	Good forecasts (PE<5%)	*Regression*	*Similarity analysis*	*Custom*
Low yield	19	4	0	2	2
High yield	17	5	0	4	1
Total	***36***	***9***	*0*	*6*	*3*

Supplementary Table 5. The month of harvest for soft wheat in the different Member States of the European Union as used in the analysis.

		Month of harvest
**Member State**		*Soft wheat*
Austria	AT	JULY
Belgium	BE	JULY
Bulgaria	BG	JULY
Czech Republic	CZ	JULY
Germany	DE	JULY
Denmark	DK	AUGUST
Estonia	EE	AUGUST
Spain	ES	JUNE
Finland	FI	AUGUST
France	FR	JULY
Greece	GR	JUNE
Hungary	HU	JULY
Ireland	IE	AUGUST
Italy	IT	JULY
Lithuania	LT	AUGUST
Luxemburg	LU	JULY
Latvia	LV	AUGUST
Netherlands	NL	JULY
Poland	PL	AUGUST
Portugal	PT	JUNE
Romania	RO	JULY
Sweden	SE	AUGUST
Slovenia	SI	JULY
Slovakia	SK	JULY
United Kingdom	UK	JULY
